# Removal of alleles by genome editing (RAGE) against deleterious load

**DOI:** 10.1186/s12711-019-0456-8

**Published:** 2019-04-17

**Authors:** Martin Johnsson, R. Chris Gaynor, Janez Jenko, Gregor Gorjanc, Dirk-Jan de Koning, John M. Hickey

**Affiliations:** 1 The Roslin Institute and Royal (Dick) School of Veterinary Studies, The University of Edinburgh, Midlothian, EH25 9RG Scotland UK; 2 Department of Animal Breeding and Genetics, Swedish University of Agricultural Sciences, Box 7023, 750 07 Uppsala, Sweden

## Abstract

**Background:**

In this paper, we simulate deleterious load in an animal breeding program, and compare the efficiency of genome editing and selection for decreasing it. Deleterious variants can be identified by bioinformatics screening methods that use sequence conservation and biological prior information about protein function. However, once deleterious variants have been identified, how can they be used in breeding?

**Results:**

We simulated a closed animal breeding population that is subject to both natural selection against deleterious load and artificial selection for a quantitative trait representing the breeding goal. Deleterious load was polygenic and was due to either codominant or recessive variants. We compared strategies for removal of deleterious alleles by genome editing (RAGE) to selection against carriers. When deleterious variants were codominant, the best strategy for prioritizing variants was to prioritize low-frequency variants. When deleterious variants were recessive, the best strategy was to prioritize variants with an intermediate frequency. Selection against carriers was inefficient when variants were codominant, but comparable to editing one variant per sire when variants were recessive.

**Conclusions:**

Genome editing of deleterious alleles reduces deleterious load, but requires the simultaneous editing of multiple deleterious variants in the same sire to be effective when deleterious variants are recessive. In the short term, selection against carriers is a possible alternative to genome editing when variants are recessive. Our results suggest that, in the future, there is the potential to use RAGE against deleterious load in animal breeding.

**Electronic supplementary material:**

The online version of this article (10.1186/s12711-019-0456-8) contains supplementary material, which is available to authorized users.

## Background

Deleterious load is an unavoidable fact of genetics that has a sizeable impact on the fitness of populations [1]. Most individuals have de novo deleterious mutations due to errors in DNA replication [2–4] and inherit many more from their ancestors. Reducing the number of deleterious variants in livestock populations could improve fitness traits, with subsequent benefits for animal welfare and profitability. In this paper, we use simulation of deleterious variants in an animal breeding program to evaluate the efficiency of genome editing and selection against carriers for improving fitness traits in livestock.

Deleterious variants can have large or small effects. Recessive lethal variants are the most obvious symptoms of large-effect deleterious mutations [5–12]. However, estimated distributions of the effects of deleterious mutations from several species indicate that most of the deleterious load is due to many variants each with a small effect [13–16]. In practice, large-effect variants that cause recessive lethality are easier to identify and manage. However, this raises the question: what can be done about polygenic deleterious load?

Deleterious variants of large and small effect can be identified by bioinformatics screening methods that use sequence conservation and biological prior information about protein function [17–21]. Such approaches have been applied to whole-genome sequence data to detect deleterious variants in crop plants [22–24], livestock [25–27], and humans [28, 29]. With the decreasing cost of genome sequencing, and the large initiatives to sequence livestock animals, we can anticipate that screening of sequence variants will become a routine part of animal breeding.

Once deleterious variants are discovered, there are two obvious ways to incorporate them into breeding programs: genome editing or selection. Genome editing is a suite of methods to modify the genomic DNA of an organism that allows not just insertion and deletion but replacement of sequences with a higher efficiency than previous methods, which involved difficult procedures such as microinjecting DNA into the nucleus of zygotes that produces engineered embryonic stem cells for implantation into chimeric embryos (reviewed by [30, 31]). Genome editing has shown theoretical promise for improving breeding progress by promoting favorable alleles [32, 33], and for managing recessive lethal variants [34]. Selection against carriers is the strategy of choice for removing monogenic recessive deleterious variants from animal breeding populations [5, 35]. Analogously, one could select against deleterious alleles at many loci by avoiding selection candidates with a high deleterious load. We regard this as a natural extension of avoiding sires that carry alleles for monogenic defects.

The aim of this paper was to compare the efficiency of genome editing and selection against carriers for decreasing deleterious load in an animal breeding program. We simulated polygenic deleterious load that is subject to natural selection in a simulation of a closed animal breeding population that is artificially selected for a quantitative performance trait representing the breeding goal. We compared removal of alleles by genome editing (RAGE) to selection against carriers using genotypes at deleterious variants. We compared strategies for prioritizing variants for editing and individuals for selection based on deleterious allele and genotype frequencies, and evaluated how they improved the fitness of the population.

## Methods

We used simulations to compare genome editing and selection against carriers using genotypes at deleterious variants. The population was similar, in terms of its size and pedigree structure, to a single breeding line of pigs. We simulated artificial selection for a quantitative trait representing the breeding goal, and natural selection for a fitness trait representing reduced probability of survival due to deleterious variants. The fitness trait was polygenic with multiplicative fitness effects and an effect size distribution that was inspired by estimates of the distribution of deleterious effects in human populations [13].

In summary, the simulations consisted of 50 replicates of:

1. coalescent process simulation to create ancestral haplotypes;
2. setting up a quantitative trait and a fitness trait;
3. 15 generations of natural selection against deleterious variants, the first five using 1000 random matings per generation and the following 10 using 500 random matings per generation;
4. 20 generations of historical breeding with natural selection and simultaneous selection on true breeding value for the breeding goal trait;
5. and finally 10 generations of future breeding, where we evaluated scenarios with genome editing or selection against carriers.

**Fig. 1:**
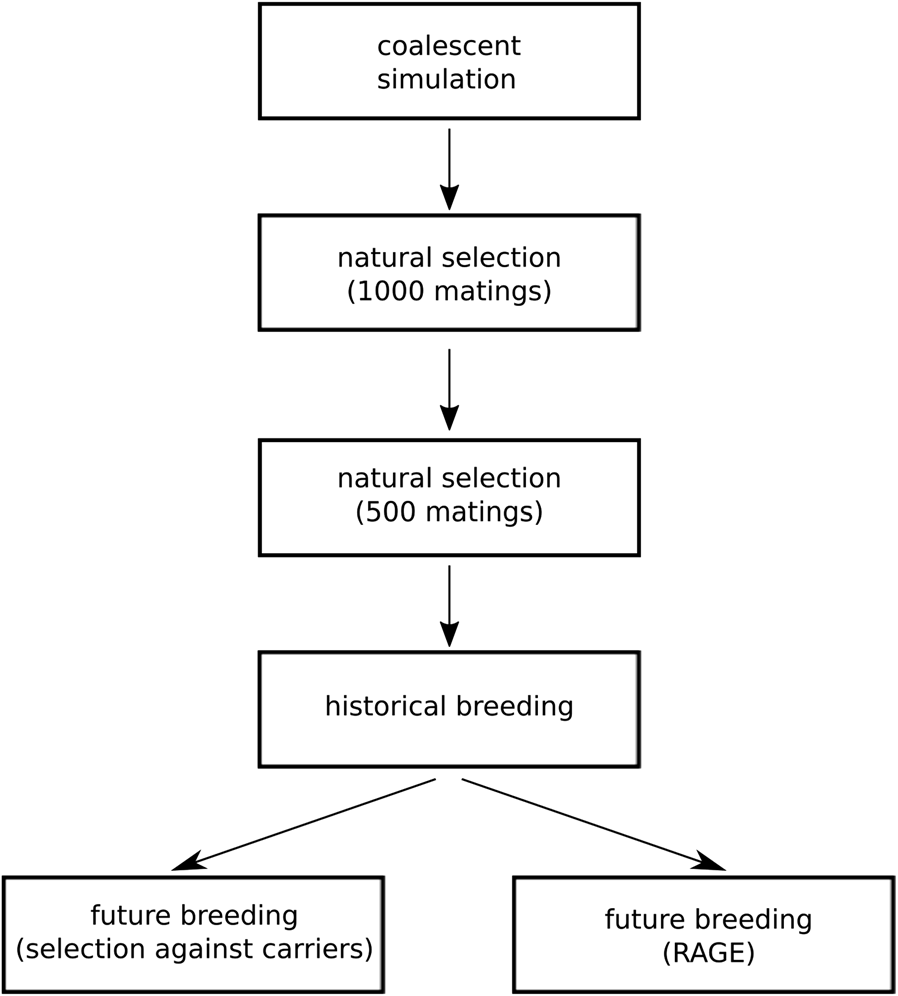
Flow chart with an overview of the simulations

Figure 1 shows an overview of this workflow. We also tested a longer (25 generations) historical breeding phase, and a shorter historical breeding phase (10 generations of natural selection instead of 15, followed by 5 generations of historical breeding).

### Simulation of whole-genome sequence data and historical evolution

We used the Markovian coalescent simulator [36] to generate ancestral haplotypes. We modelled a genome consisting of 10 chromosomes each one Morgan long with 6.75 × 10^8^ bp. The chromosomes were simulated using a mutation rate of 1.6 × 10^−8^ per site, and an effective population size that changed over time to reach a final size of 100. The effective population size was set to be 10^6^ at 190,000 generations ago, 100,000 at 100,000 generations ago, and 100 at current time, with linear decreases (on the 4 × N_e_ × time scale) in between. We also tested the simulation of founder haplotypes by using a constant effective population size of 100 individuals.

### Simulation of quantitative and fitness traits

To capture artificial selection for the breeding goal and natural selection against deleterious variants simultaneously, we modelled a quantitative breeding goal trait and a fitness trait.

The breeding goal trait was a polygenic quantitative trait with additive effects. We randomly assigned 10,000 segregating sites (1000 per chromosome) as quantitative trait variants for the breeding goal trait with additive effects drawn from a normal distribution. We also tested drawing the additive effects from a gamma distribution, and then randomly choosing a sign with equal probability of a positive and a negative effect. We used a shape parameter of 11, based on the estimate for pigs from [37].

Fitness was a polygenic multiplicative trait that represented probability of survival prior to artificial selection. We randomly assigned 10,000 segregating sites as fitness variants (again 1000 per chromosome), choosing variants that had allele frequencies lower than 0.01 in scenarios in which variants were codominant, and 0.1 in scenarios in which variants were recessive. The fitness variants were chosen independently of the quantitative trait variants. The deleterious effect size was expressed as a selection coefficient *s* against the mutant allele, ranging from 0 (no deleterious effect) to 1 (a lethal allele). The fitness of each genotype was 1 for the homozygous wildtype, $1 {-} hs$ for the heterozygote, and $1 {-} s$ for the mutant homozygote, where *h* is a dominance coefficient. Dominance coefficients were either 0 for recessive variants or 0.5 codominant variants. We assumed multiplicative effects, so that the fitness of an individual was the product of the contribution of each fitness variant. The effect sizes were drawn from a mixture of three uniform distributions with one-third of the variants being small (0 < *s* < 10^−4^), one-third intermediate (10^−4^ < *s* < 0.1), and one-third large (0.1 < *s* < 1). These proportions were chosen based on the estimated distribution of deleterious effects in humans [13].

Deleterious mutations occurred randomly during natural selection and historical breeding with a mutation rate of 10^−4^ per locus, to give a deleterious mutation rate of 1 per individual and genome. This is a conservative estimate for the deleterious mutation rate in mammals. No back-mutation was allowed, meaning that only wild type alleles could mutate. Quantitative trait variants for the breeding goal trait did not mutate, except during the initial coalescent simulation to create ancestral haplotypes.

### Pedigree structure and selection for the breeding goal trait

At each generation during the historical and future breeding, we first applied natural selection for fitness, then artificial selection for the breeding goal trait on the remaining individuals. For natural selection, we drew a uniformly distributed random number between 0 and 1 for each individual. If the number was larger than the fitness value for that individual, the individual was removed from the population before selection. For artificial selection, we selected 25 sires and a variable number of dams based on true breeding value for the breeding goal trait. Mating between sires and dams was random. Each dam had 10 progeny. We selected the number of dams required to reach a population of 5000 individuals at the average level of deleterious load at the start of historical breeding.

### Deleterious variant discovery

To simulate the discovery of deleterious variants, we selected a random fraction of the deleterious variants that segregated at the end of historical breeding and assumed them to be discovered. We used a discovery rate of 0.75 for the main scenarios, but also tested discovery rates of 0.1, 0.5 and 1. To simulate imperfect detection of deleterious variants, we chose neutral segregating variants as false positives at random. We added false positives so that the total number of variants detected was equal to the number of segregating deleterious variants, and if discovery rate was *d*, a fraction 1 − *d* were false positives. These discovered variants were allowed to be edited or used for selection against carriers subsequently.

### Genome editing

For the future breeding scenarios that used removal of alleles by genome editing, we simulated editing of discovered deleterious variants in selected sires. That is, first we selected sires on the breeding goal trait, and then applied genome editing for the fitness trait to all the 25 sires. For variants for which a sire was not already homozygous wild type, we edited the genotype to homozygous wild type, until a set number of variants had been edited. We assumed that editing was accurate, such that it always produced wild type homozygotes, and had no deleterious off-target effects. We edited 1, 5, or 20 variants per sire. We only edited variants that were segregating in the population.

### Mortality

To simulate mortality during genome editing, we randomly removed edited sires, according to a given mortality rate, and replaced them with lower-ranked candidate sires from the population. The replacement sires were not genome-edited. We applied no mortality rate for the main scenarios, but also tested mortality rates of 0.1, 0.25, and 0.5. The mortality rate parameter represents both direct mortality during the editing process, and also failures of editing that would introduce unwanted, presumably deleterious, alleles and lead to culling the sire.

### Scenarios

#### Removal of alleles by genome editing (RAGE)

During future breeding, we removed alleles by genome editing at discovered deleterious variants in selected sires. We used five strategies for prioritizing variants for editing. These strategies were based on information that would be available from genotyping the sires at discovered deleterious variants, namely the deleterious allele and genotype frequencies. We assumed that the deleterious effect size was unknown. The strategies were:

- Based on high frequency, removing variants in decreasing order of deleterious allele frequency. The rationale for this strategy was that recessive deleterious variants cause more damage when they are common, and therefore removing high-frequency variants, first, might be beneficial.
- Based on low frequency, removing variants in increasing order of deleterious allele frequency. The rationale for this strategy was that since deleterious variants are removed by natural selection, low-frequency variants are more likely to be damaging.
- Based on lack of homozygotes, removing variants in decreasing order of the difference between observed and expected deleterious allele homozygotes. The rationale for this strategy was that scanning for a deficit of homozygotes is a way to detect recessive lethal individuals [6], and might therefore help identify variants that are more damaging.
- Based on intermediate frequency, removing variants in decreasing order of deleterious allele frequency after applying a threshold to exclude variants with an allele frequency higher than 0.25. The rationale for this strategy was to remove recessive variants that are common, while filtering out variants with allele frequencies that are too high to have large negative effects.
- Random, in random order, using the same random order for all sires. The rationale for this strategy was to serve as a control.

For comparison, we also ran a baseline scenario without genome editing, starting from the same initial populations after historical breeding.

#### Selection against carriers

During future breeding, we performed selection against carriers in sires by identifying carriers with a high deleterious load and removing them before selection. We avoided the 100, 250, or 500 individuals with the highest load when selecting sires.

We used three strategies for selecting carriers. These strategies were based on information that would be available from genotyping the sires at discovered deleterious variants, namely the deleterious allele frequencies, genotype frequencies, and individual numbers of deleterious alleles. The strategies were:

- Total load, avoiding individuals that carry the largest number of deleterious alleles, summing over the discovered variants. The rationale for this strategy was to use all the available information for selection.
- Heterozygous load, avoiding individuals that carry the largest number of deleterious alleles in the heterozygous state. The rationale for this strategy was that focusing on heterozygotes might be beneficial, because large-effect deleterious variants are rarely homozygous.
- Homozygous load, avoiding individuals that carry the largest number of deleterious alleles in the homozygous state. The rationale for this strategy was to serve as a control.

For comparison, we also ran a baseline scenario without selection against carriers, starting from the same initial populations after historical breeding.

### Metrics and statistical analysis

We evaluated the simulated scenarios by the improvement in average fitness of the individuals in the population. By an individual’s fitness, we mean the genetic value for the fitness trait. We calculated the average change in fitness from the first to the tenth generation of future breeding, and compared it to the change in fitness in a baseline condition without genome editing and selection against carriers, reporting mean and standard error of the mean.

We evaluated the effect of total number of fitness variants in the genome and their dominance coefficients on the number of segregating variants, the frequencies of deleterious alleles, and the deleterious load. By number of segregating variants, we mean the number of fitness variants that remained variable in the population after natural selection and historical breeding. By deleterious load, we mean the number of deleterious alleles that are carried by an individual. When considered separately, heterozygous load means the number of deleterious alleles that are carried in the heterozygous state, and homozygous load means the number of deleterious alleles that are carried in the homozygous state.

We performed simulations using AlphaSimR which was modified to allow for fitness traits. AlphaSimR runs on the R statistical environment [38], and uses Rcpp and Armadillo [39–41]. We calculated summary statistics in the R statistical environment, and made graphs with ggplot2 [42]. The simulation scripts are available from https://bitbucket.org/hickeyjohnteam/rage/.

## Results

We simulated a closed animal breeding population under selection for a breeding goal trait, which was affected simultaneously by deleterious load consisting of either codominant or recessive variants. Our results show that both genome editing of deleterious alleles and selection against carriers can reduce deleterious load in some cases, but is inefficient at reducing it in other cases. The efficiency of genome editing and selection against carriers, and which variant prioritization strategy is the most efficient, depend on whether the deleterious variants are codominant or recessive.

### Deleterious allele frequencies and load in simulated populations

**Fig. 2:**
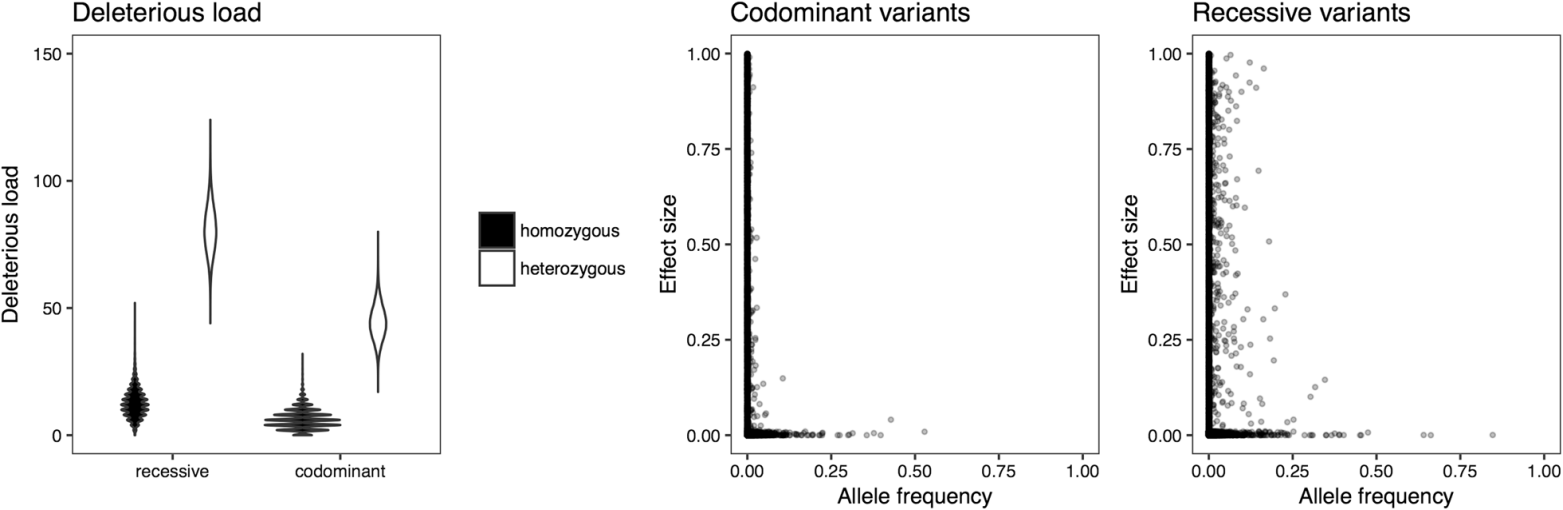
Deleterious allele frequencies and load in simulated populations when deleterious variants are either codominant or recessive. The violin plot shows individual deleterious load broken down into heterozygous and homozygous load with codominant or recessive variants. The scatterplots show the relationship between deleterious allele frequency and deleterious effect size. The effect size ranges from 0 to 1, where an effect size of 0 means a harmless variant, and of 1 a lethal variant (see “Methods”)

The simulated populations had on average 4444 (standard deviation SD = 217) segregating deleterious variants in the codominant case, and 3634 (SD = 177) in the recessive case. Each individual carried on average a load of 52 (SD = 7.6) deleterious alleles in the codominant case and 89 (SD = 9.7) deleterious alleles in the recessive case. Figure 2 shows violin plots of the deleterious load carried by individuals at both levels of dominance. The distribution of deleterious alleles was affected by dominance. The scatterplots in Fig. 2 show the relationship between effect size and frequency of deleterious alleles after historical breeding. When deleterious variants were codominant, most deleterious variants were rare. When deleterious variants were recessive, there were more deleterious alleles that included even large-effect variants at intermediate frequencies, which are candidates for removal by genome editing.

We also tested varying population parameters to assess the sensitivity to assumptions. We varied the total number of fitness variants in the genome, tested a breeding goal trait with gamma-distributed effects rather than normally-distributed effects, and varied the length of the historical breeding phase. The resulting average fitness and the distribution of deleterious allele frequencies and load were broadly similar (see Additional file 1: Table S1, Additional file 2: Figure S1 and Additional file 3: Figure S2). The exception was in the case of a shorter historical breeding, which resulted in lower deleterious allele frequencies and load.

### Comparison of RAGE and selection against carriers

**Fig. 3:**
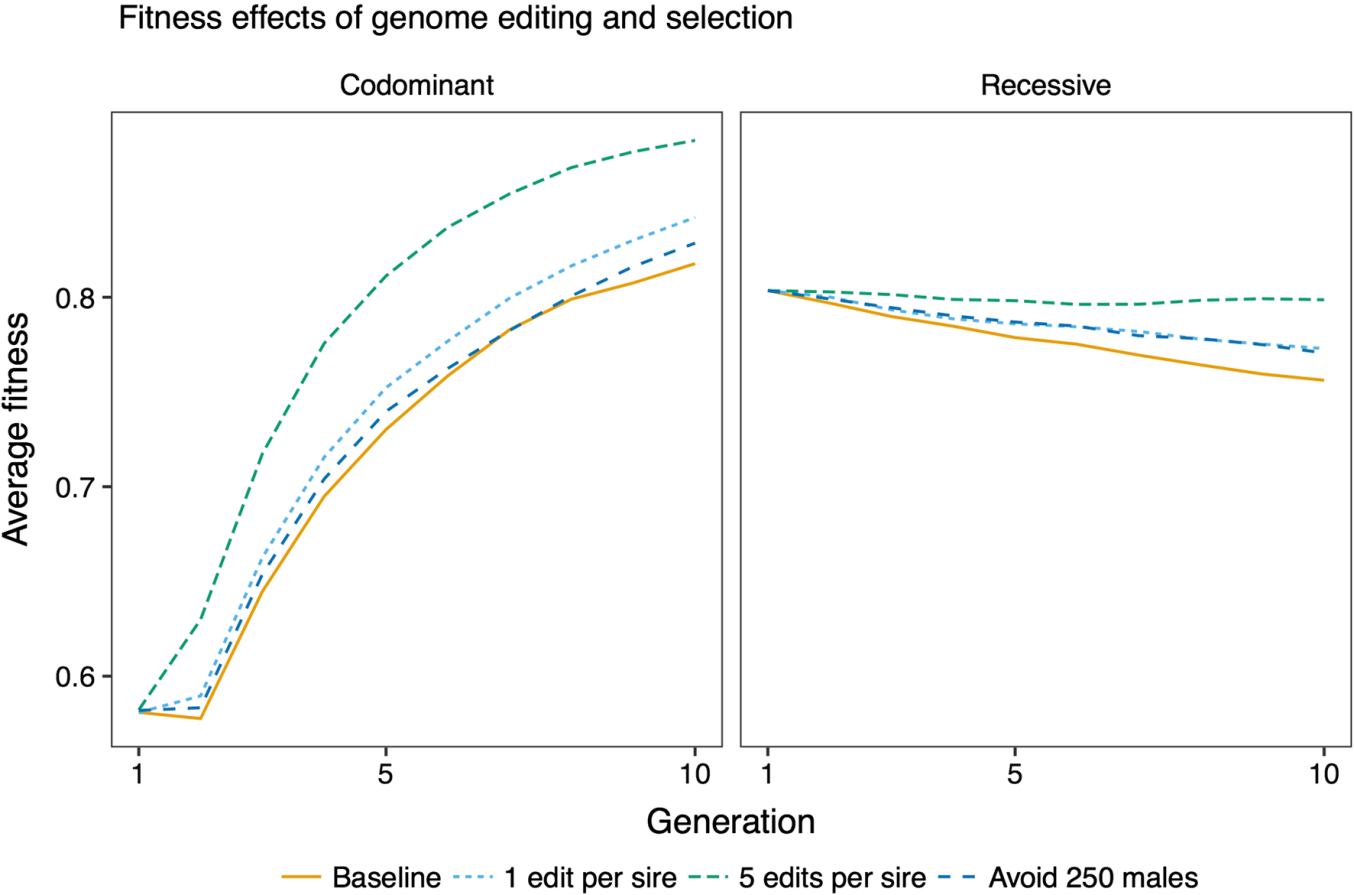
Comparison of the effect on average fitness of genome editing and selection against carriers, using the best-performing editing strategies for each dominance level. The baseline condition is selection for the breeding goal trait with no effort to reduce deleterious load. The discovery rate was 0.75, meaning that 75% of the deleterious variants that segregated after historical breeding were discovered and could be edited. The lines show averages across 50 replicates

**Fig. 4:**
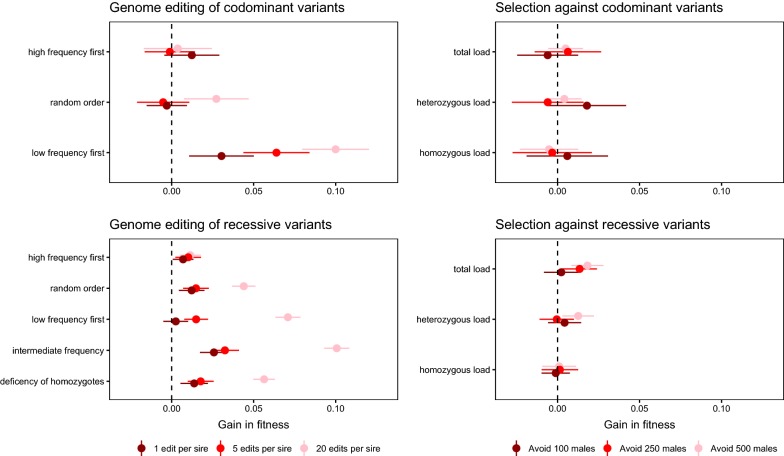
Effect on fitness of removal of deleterious alleles by genome editing and selection against carriers. The points show the mean change in fitness over ten generations compared to the baseline case of breeding without editing or selection, varying the number of edits per sire or the number of males avoided, and the strategy for variant prioritization or selection. The error bars are 2 standard errors of the mean

The difference in the distribution of deleterious allele frequencies induced by codominant and recessive variants translates to differences in the efficiency of RAGE and selection against carriers. Figure 3 shows a comparison of genome editing using the best-performing variant prioritization strategy, and selection against carriers using total deleterious load. When deleterious variants were codominant, the best-performing strategy prioritized low-frequency variants for removal by editing, and selection against carriers was inefficient. When deleterious variants were recessive, the best-performing strategy prioritized variants with an intermediate frequency for removal by editing, and selection against carriers was comparable to genome editing of one variant per sire. However, multiplex editing of five variants per sire outperformed selection against carriers. Figure 4 shows the change in fitness under different scenarios after 10 generations of future breeding, compared to the baseline case of breeding without genome editing or selection against carriers. In summary, selection against carriers was effective only against recessive deleterious variants, whereas genome editing could be effective at both levels of dominance, but with different variant prioritization strategies performing the best.

**Fig. 5:**
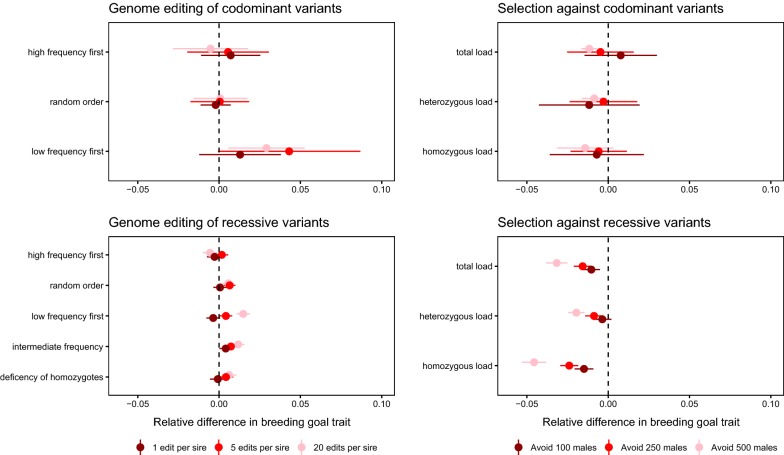
Effect on the breeding goal trait of removal of deleterious alleles by genome editing and selection against carriers. The points show the mean relative change in the breeding goal trait over ten generations compared to the baseline case of breeding without editing or selection, expressed as the fraction of increase without genome editing or selection against carriers. The error bars are 2 standard errors of the mean

RAGE tended to improve or have no effect on the genetic gain of the breeding goal trait, whereas selection against carriers decreased genetic gain of the breeding goal trait. In scenarios in which deleterious load was alleviated, genetic gain of the breeding goal trait increased up to 4% with RAGE, and in scenarios with selection against carriers, genetic gain in the breeding goal trait decreased by up to 5%. Figure 5 shows the relative change in genetic gain of the breeding goal trait compared to the baseline scenario of no editing or selection against carriers. Our model allowed population size to fluctuate with deleterious load, and scenarios using selection against carriers reduced the male population even more by excluding individuals with a high deleterious load. Thus, genome editing makes it possible to improve fitness traits without sacrificing selection intensity of the breeding goal.

In the next paragraphs, we present first the effects of different variant prioritization strategies on RAGE, then the effect of selection strategies on selection against carriers, and finally the impact of the ability to detect deleterious variants accurately.

### Effect of variant prioritization strategy on RAGE

**Fig. 6:**
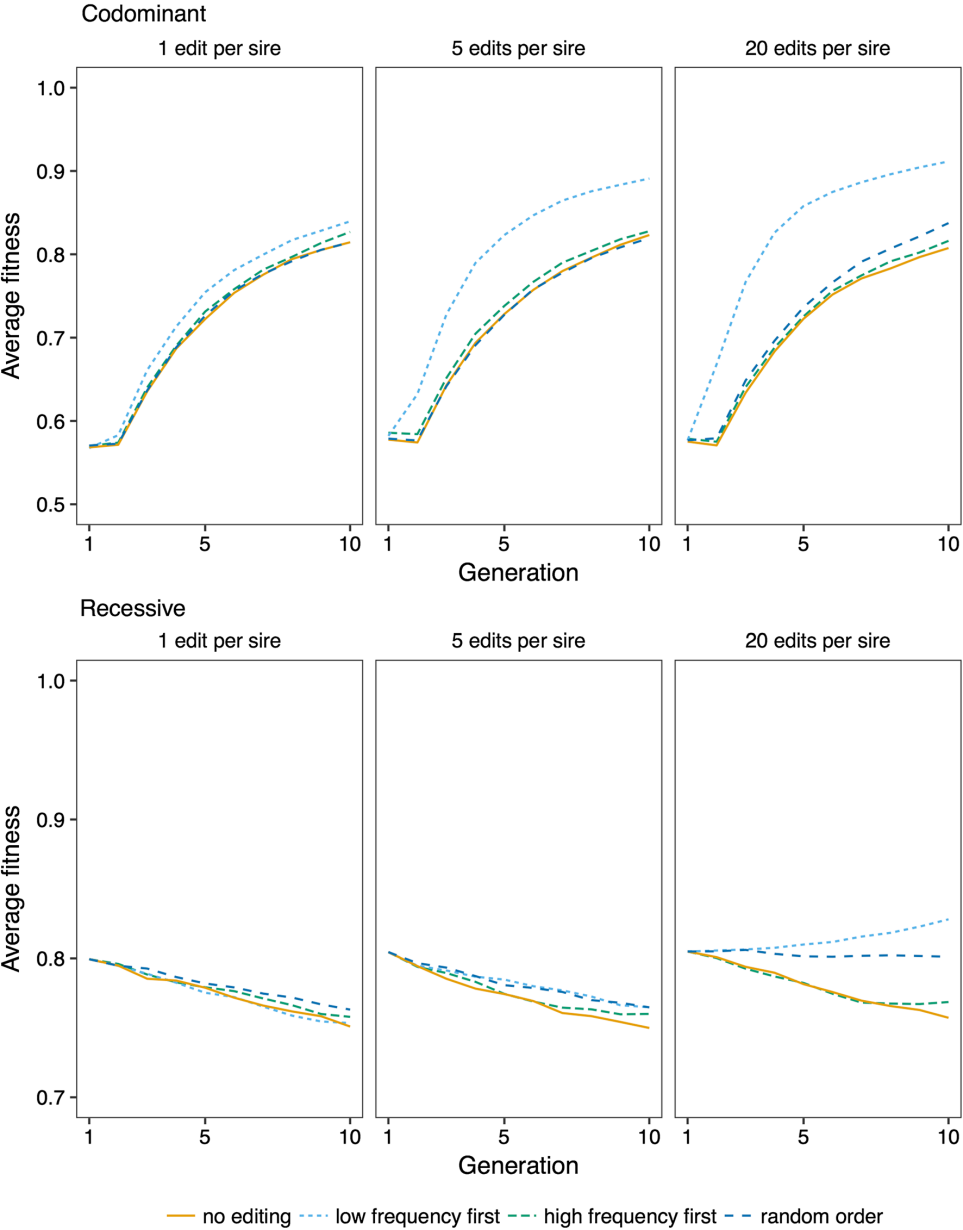
Effect of genome editing on fitness. Average fitness over ten generations of future breeding with different editing strategies, and editing of 1, 5, or 20 variants per sire. The discovery rate was 0.75. The lines show averages across 50 replicates

**Fig. 7:**
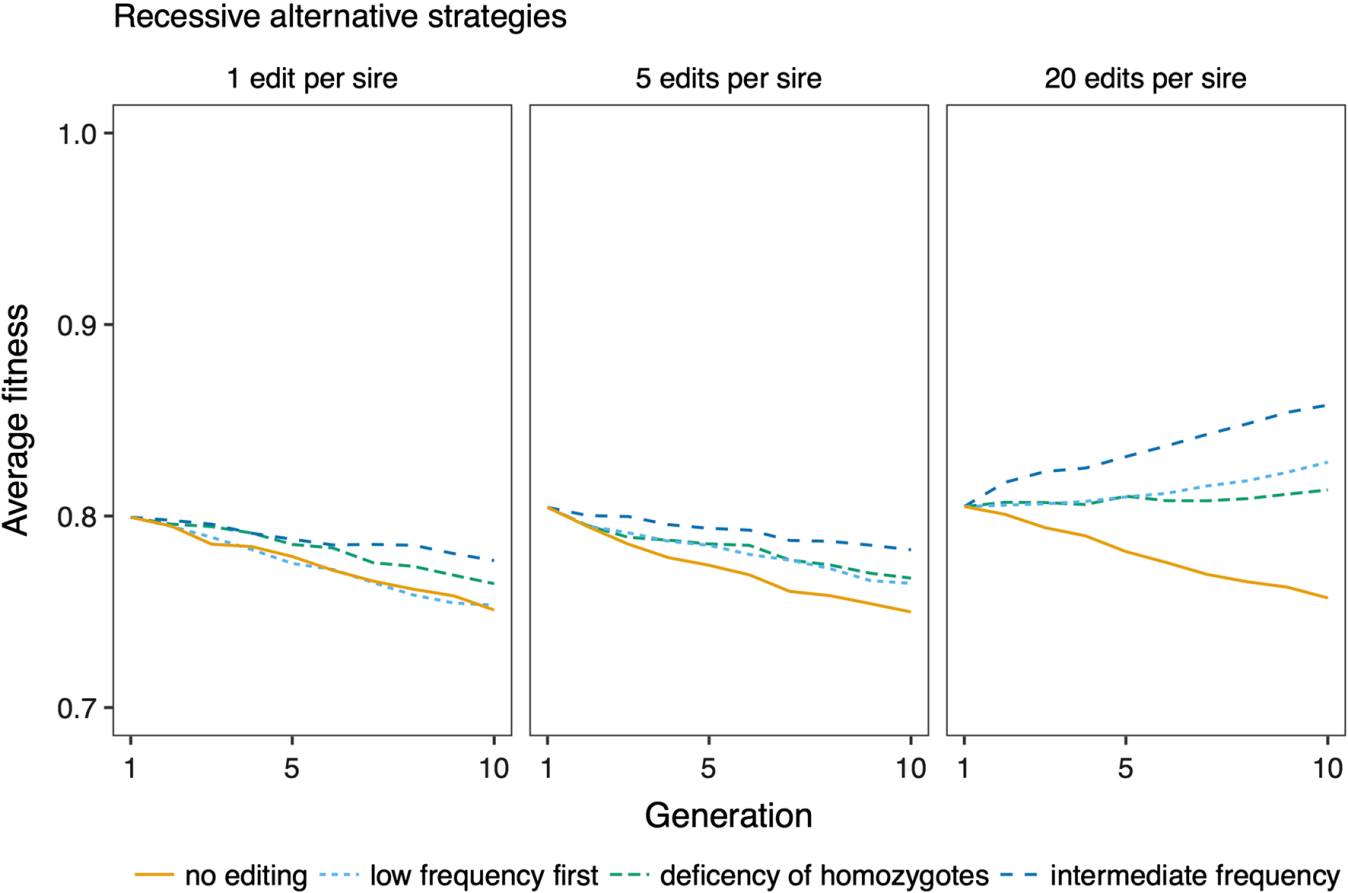
Effect of prioritizing variants with intermediate allele frequency, and in order of their deviation from expected homozygosity. Average fitness over ten generations of future breeding with 10,000 recessive deleterious variants, editing 1, 5, or 20 variants per sire. The discovery rate was 0.75. The lines show averages across 50 replicates

The efficiency of genome editing for improving fitness was affected by the number of variants edited per sire, and the strategy for prioritizing variants for editing. Figure 6 shows trajectories of fitness across generations of genome editing, by varying the number of variants edited, and by prioritizing variants at low frequency, variants at high frequency, or randomly chosen deleterious variants for editing. Figure 7 shows the trajectories of fitness during future breeding using variant prioritization strategies that were devised for recessive variants: prioritizing variants with an intermediate frequency by applying an allele frequency threshold of 0.25, and editing variants based on their deficit of homozygotes. For both levels of dominance, fitness improved more by prioritizing low-frequency variants for editing than by editing in random order, or prioritizing high-frequency variants. When variants were recessive, fitness was most improved by prioritizing variants with an intermediate allele frequency. Prioritizing variants with a deficit of homozygotes did not improve efficiency compared to prioritizing variants with an intermediate allele frequency.

The variant prioritization strategies also differed in how many distinct variants were edited. Table 1 shows the average number of distinct variants edited during 10 generations of future breeding with genome editing. Prioritizing low-frequency variants for editing resulted in the largest number of distinct variants being edited, using random order and intermediate allele frequency strategies resulted in an intermediate number of distinct variants being edited, and prioritizing high-frequency variants for editing led to the smallest number of distinct variants being edited. Thus, when variants were codominant, the greatest improvement in fitness came from editing rare large-effect variants carried by few individuals, but when variants were recessive, the greatest improvement came from removing a relatively smaller number of deleterious variants with an intermediate frequency.

**Table 1:** Number of distinct variants edited over 10 generations of future breeding with different strategies

Strategy	1 edit per sire	5 edits per sire	20 edits per sire
Codominant variants			
Low frequency	179	646	995
High frequency	9	45	184
Random order	34	133	431
Recessive variants			
Low frequency	175	685	1111
High frequency	9	45	186
Random order	37	148	467
Deficiency of homozygotes	21	71	217
Intermediate frequency	45	144	392

The numbers are averages across 50 replicates

### Effect of selection strategy on selection against carriers

The efficiency of selection strategies against carriers also varied with the number of males that were avoided, selection strategy, and dominance (see Additional file 4: Figure S3). When deleterious variants were codominant, selection against carriers was inefficient regardless of the strategy. When deleterious variants were recessive, selection on total load was the most efficient selection strategy. In no case was selection on only heterozygous or homozygous load better.

### Effect of the ability to discover deleterious variants

**Fig. 8:**
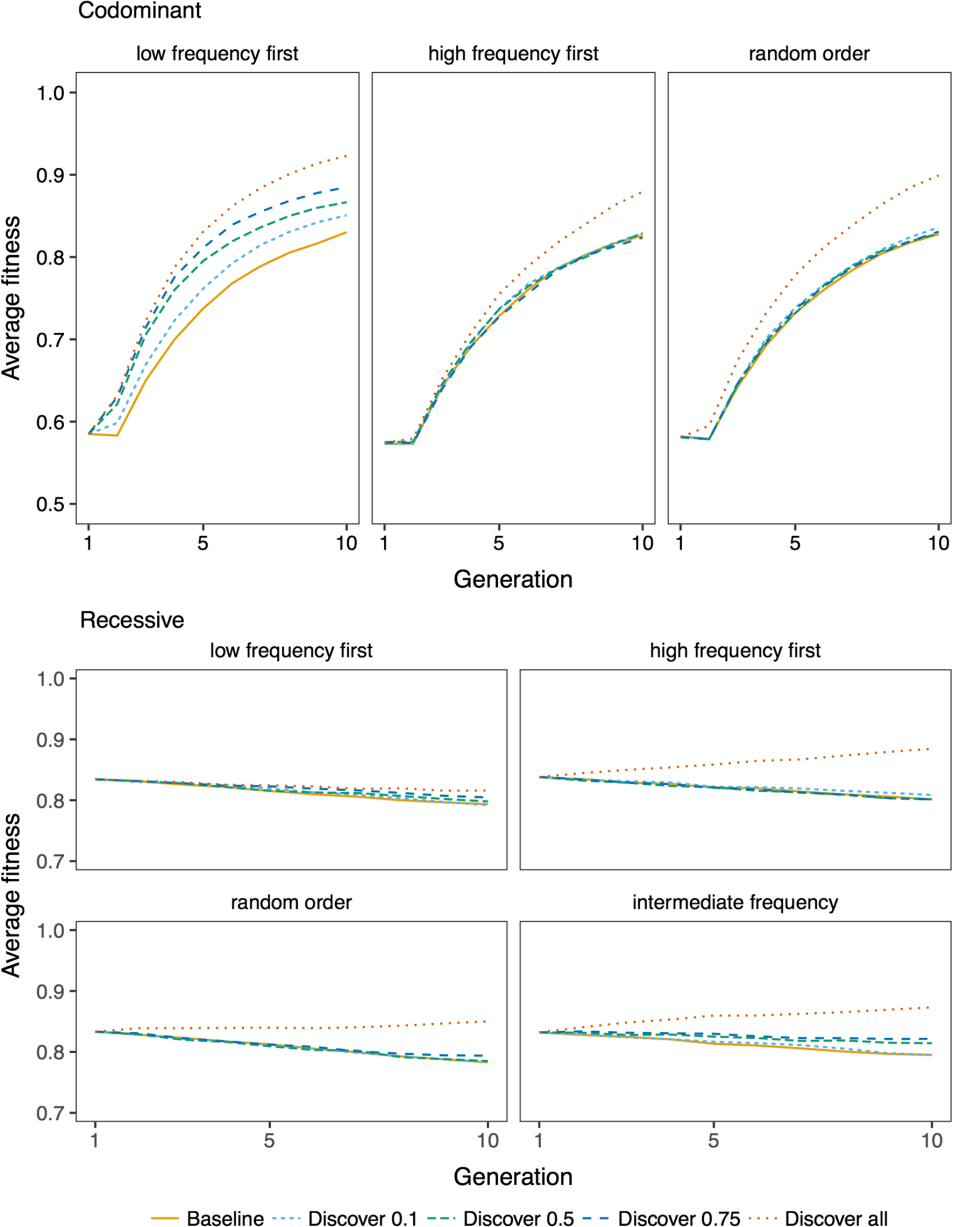
Effect of discovery rate. Average fitness over ten generations of future breeding with 10,000 deleterious variants, 5 edited variants per sire, and discovery rates of 0.1, 0.5, 0.75, and 1. The lines show averages across 50 replicates. The baseline scenario is with no editing

The efficiency of genome editing and the relative performance of variant prioritization strategies was affected by the discovery rate. Figure 8 shows fitness trajectories by varying how many of the deleterious variants could be discovered and edited. Concentrating on the best-performing strategies, when deleterious variants were codominant and low-frequency variants were prioritized, fitness improvement increased with discovery rate. When deleterious variants were recessive and variants with an intermediate frequency were prioritized, editing was inefficient when discovery rate was low (0.1), but there was little difference between a discovery rate of 0.5 and 0.75. Thus, RAGE was susceptible to false positives, but when variants were recessive, whether the false positives made up 25% or 50% of the detected variants had less impact.

**Fig. 9:**
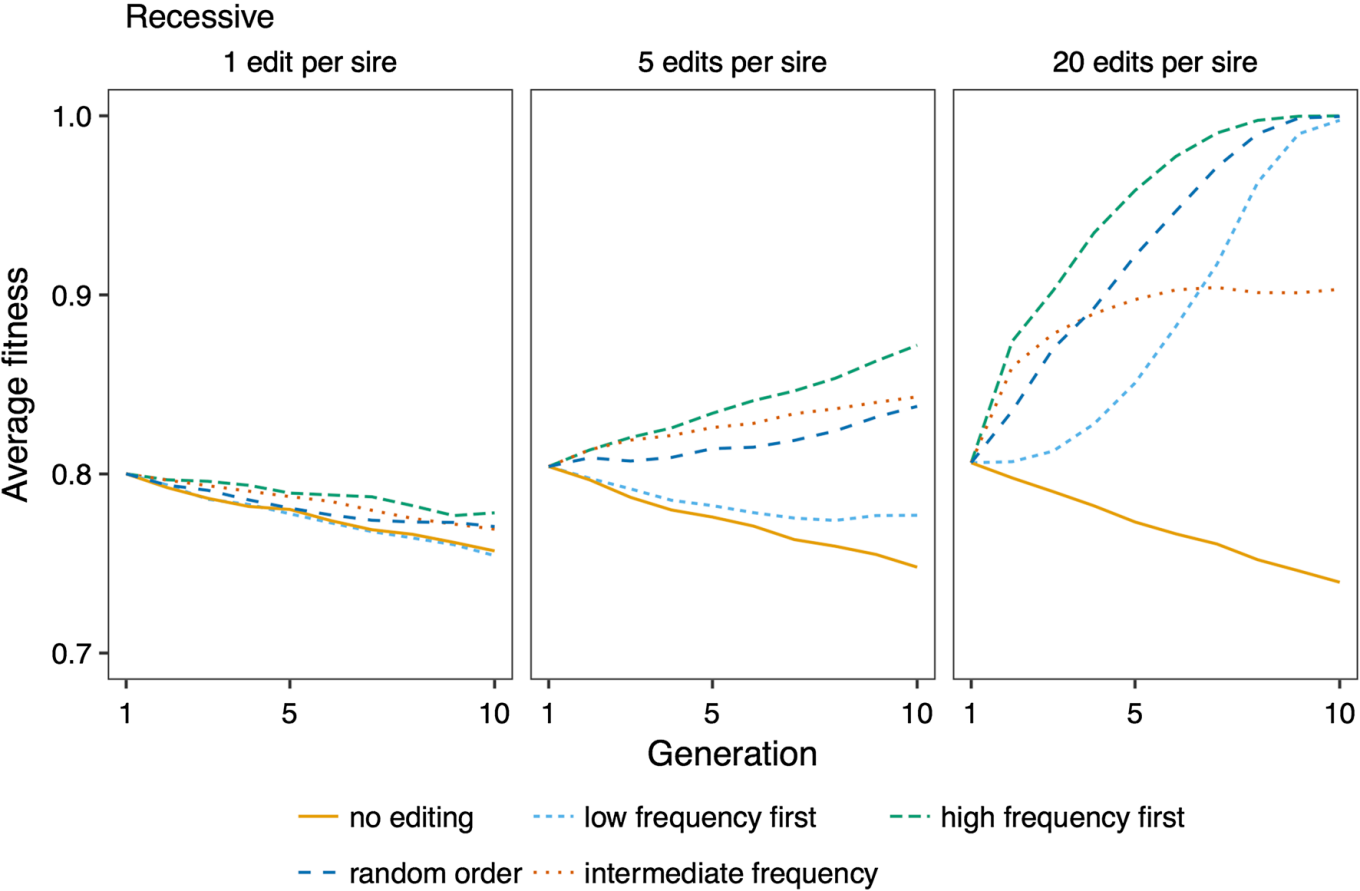
Relative efficiency of genome editing strategies against recessive deleterious variants when variant discovery is perfect (discovery rate is 1). The lines show averages across 50 replicates

When variants were recessive, a high discovery rate changed the relative ranking of variant prioritization strategies, making the high-frequency strategy more efficient than the intermediate-frequency strategy, which was not the case at lower discovery rates. To illustrate this, Fig. 9 shows fitness trajectories when all segregating deleterious variants were discovered with no false positives (i.e., a discovery rate of 1). Taken together, this means that the presence of false positives affects the strategies that use allele frequency information for variant prioritization, differently.

### Effect of additional mortality during editing

**Fig. 10:**
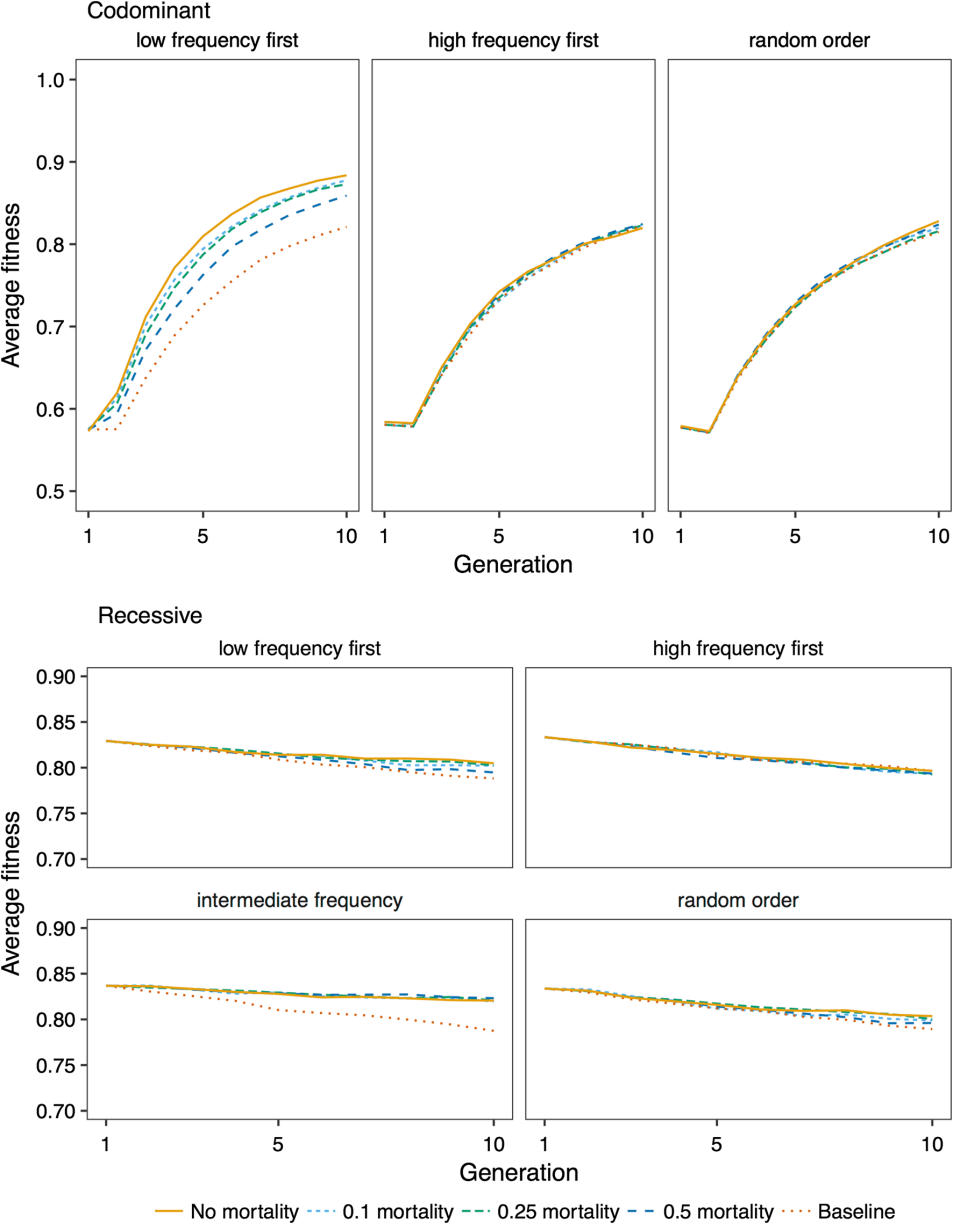
Effect of mortality due to editing on fitness. Average fitness over ten generations of future breeding with 10,000 deleterious variants, 5 edited variants per sire, a discovery rate of 0.75, and mortality rates of 0, 0.1, 0.25, and 0.5. The lines show averages across 50 replicates. The baseline scenario is with no editing

The effect of additional mortality during editing on fitness improvement was also affected by dominance. Figure 10 shows fitness trajectories by varying mortality rate during editing. When deleterious variants were codominant and low-frequency variants were prioritized, fitness improvement decreased with mortality. However, when deleterious variants were recessive and variants with an intermediate frequency were prioritized, there was little effect of additional mortality on the fitness improvement.

## Discussion

In this paper, we simulated deleterious load in an animal breeding program, and compared the efficiency of genome editing and selection for decreasing it. We found that both removal of alleles by genome editing and selection against carriers can reduce deleterious load in some scenarios. Dominance of deleterious variants affects the efficiency of genome editing and selection against carriers, and determines which variant prioritization and selection strategy are the most efficient. In the light of these results, we discuss (1) deleterious load in animal breeding populations, (2) the efficiency of different variant prioritization and selection strategies, (3) the factors that improve the efficiency of genome editing of deleterious variants, (4) the assumptions that underlie the simulations, and (5) the implications for applications of RAGE and selection against carriers in breeding.

### Deleterious load in animal breeding populations

The deleterious loads in the simulated populations were comparable to the observed loads of putative loss-of-function variants in mammals, but they were much smaller than the numbers of deleterious single nucleotide variants predicted with sequence bioinformatics-based methods. Humans are estimated to carry an average load of around 100 [43, 44] or 150 [45] putative loss-of-function variants in protein-coding genes. The average load of loss-of-function variants observed in cattle is 65 [46]. The load of deleterious variants in our simulations (around 90 deleterious alleles per individual when variants were recessive) was comparable to these numbers. However, observed loads of deleterious nonsynonymous single nucleotide variants that are predicted by bioinformatics methods are much larger: i.e. ranging from 300 to 800 in humans [47, 48], and ~ 656 in a pig population [49]. This suggests that our assumptions about the distribution of the size of the effect of deleterious variants or genomic deleterious mutation rate may be conservative, and the actual deleterious load may be larger.

### Efficiency of different variant prioritization and selection strategies

Dominance of deleterious variants affected the distribution of allele frequencies of deleterious alleles, and therefore determined which variant prioritization strategy and selection strategy was the most efficient.

When deleterious variants were codominant, large-effect deleterious variants were rare. Because codominant deleterious alleles are expressed even if they are in the heterozygous state, they are more exposed to purifying selection and their frequency decreases more quickly than that of recessive variants (which is consistent with results from deterministic single-locus population genetic models [50]). Therefore, the best variant prioritization strategy was to prioritize low-frequency variants for editing.

In contrast, when deleterious variants were recessive, there were substantial numbers of large-effect variants at intermediate frequencies. This happens because of the inefficiency of natural selection against recessive variants. Because recessive deleterious variants are expressed only if they are in the homozygous state, the more common they are the more likely they are to cause damage. In spite of this, in the presence of false positives, the best variant prioritization strategy was to prioritize variants with an intermediate frequency for editing. Because false positives are neutral variants, on average they will have higher frequencies than genuine deleterious variants. Therefore, the high-frequency variant prioritization strategy is especially susceptible to false positives. Prioritizing variants with an intermediate frequency for editing balances these effects, at least when the number of edits per sire is small. On the one hand, it avoids false positives by excluding variants at frequencies that are implausibly high for large-effect deleterious variants. On the other hand, among the remaining variants, it prioritizes high-frequency variants, which, when variants are recessive, cause more damage. Another benefit of prioritizing intermediate-frequency variants, or randomly selected variants, for editing is that this strategy requires fewer distinct variants to be edited, and therefore fewer proven editing constructs to be developed and tested, compared to prioritizing low-frequency variants.

Our simulations showed no benefit from prioritizing recessive variants for editing based on a deficit of homozygotes. The strategy was inspired by the method of VanRaden et al. [6] to discover recessive lethal haplotypes, who compared the number of observed and expected homozygotes. This and related methods have been successfully used to detect recessive lethal haplotypes in livestock. Its failure as a variant prioritization method may be because the simulated deleterious variants had variable effects, most of these having each a small effect or because many variants were rare and thus led to small numbers of expected homozygotes. Furthermore, the extremely widespread use of a few sires in cattle populations [6] may give more power to detect deficits in homozygosity.

In real populations, we should expect deleterious variants that persist to be at least partially recessive, as suggested by studies of model organisms [51–53], and the ubiquity of inbreeding depression and heterosis [54, 55]. Therefore, our results suggest that we should prioritize the editing of deleterious variants with intermediate frequencies, or avoid carrier males based on their total deleterious load.

### Factors that will improve the efficiency of RAGE

According to our simulations, the most important factor for increasing the efficiency of the removal of deleterious alleles by genome editing is the ability to edit multiple variants per individual. Currently, it is not possible to produce germline-edited livestock with multiple alleles edited, but genome editing technologies are progressing rapidly. Multiplex genome editing has been performed in cells and model organisms [56–60].

The ability to accurately discover deleterious variants is also important. Methods for detecting deleterious variants predict variant consequences (e.g., stop codons and frame shifts) based on the genetic code [61, 62], or measure evolutionary conservation or constraint in multiple sequence alignments [17, 19, 20], or train statistical models to classify variants based on known deleterious variants and various predictors, possibly including variant consequences and evolutionary constraint, or functional genomic and protein structure data [18, 21, 28, 63, 64]. We expect the latter approach to become more accurate as machine-learning methods improve and as access to continually larger datasets of genetic variants and genomic data increases, and thus, it will be possible to train models on livestock rather than human data.

Additional mortality during editing decreased the efficiency of RAGE when deleterious variants were codominant, but, perhaps unexpectedly, had little impact on fitness improvement when deleterious variants were recessive. The explanation is that when a sire to be edited is lost and replaced, the replacement sire is unlikely to be also a carrier for the deleterious variant in question. However, since it will be necessary to fall back on sires with lower breeding values, genetic gain will decrease and there will be potentially be some time lag. Across 10 generations, a 50% mortality rate would lead to the cumulative loss of 125 sires, on average. Given that editing would be done in vitro, this number includes discarded embryos that carry failed genome edits, which means that this would not actually amount to the culling of that many sires with potentially impaired welfare, but high mortality rate would still be costly and problematic. The development of methods to safely and efficiently perform multiplex editing of embryos will be important for future implementation in practice.

Our simulations assumed that there was no information on the effect size of the predicted deleterious variants. This is a conservative assumption because, in fact, it may be possible to stratify predicted deleterious variants by predicted impact. For example, protein-coding variants are likely to have larger effect sizes than non-coding variants [65, 66], and loss-of-function variants are likely to have larger effect sizes than nonsynonymous single nucleotide variants. Recombination rate variation may also impact variant prioritization. In regions of low recombination rate, such as pericentromeric regions and sex chromosomes, selection against deleterious variants is less efficient due to Hill–Robertson interference [67]. This phenomenon may lead both to accumulation of deleterious variants and reduced selection for beneficial variants that are located there. Therefore, it may also be beneficial to prioritize variants in regions of low recombination rate for genome editing [68, 69].

### Assumptions underlying the simulations

Assumptions about the genetic architecture of deleterious load in these simulations include: the number of fitness variants in the genome, independent genetic architectures of the breeding goal trait and fitness, a genomic deleterious mutation rate of 1, and equal dominance coefficients for all variants. In real genomes, we expect that many more than 10,000 sites can give rise to deleterious mutations, but since the number of segregating variants was little affected by the total number of fitness variants in the genome, this assumption seems to have little impact on the resulting distribution of fitness, load, and deleterious allele frequencies. Similarly, using a gamma distribution for the breeding goal trait, thus allowing for quantitative trait loci with larger effects, did not have a major effect on the distribution of deleterious variants. However, shortening the historical breeding period led to lower frequencies of recessive deleterious variants, since the variants have less time to drift to intermediate frequencies. We simulated fitness as independent of the selected performance trait. In real populations, we expect that fitness is, to some extent, already part of the breeding goal in the form of survival, fecundity, and health traits. The level of correlation between fitness traits and the breeding goal will depend on both the genetic architecture of fitness traits and the purpose of the breeding line. This might affect the level of load within populations, but also means that it will be possible to validate deleterious variants by phenotypic means, and to include them in genomic selection models [24]. We assumed a genomic deleterious mutation rate of 1, but this is a conservative estimate, given that deleterious mutation rates for humans are often estimated to be higher (e.g., 1.6–3) [2–4]. We assumed equal dominance coefficients for all variants: either 0.5 (codominant) or 0 (recessive). In real populations, there could be a range of dominance coefficients, but recessive variants are expected to persist longer in the population.

We assumed that it was possible to apply genome editing to sires after selection, so that we could select and edit only the top sires, and have the edits be transmitted to their offspring. As discussed by Bastiaansen et al. [33], this may be achieved through cloning the top sires, or by a procedure that combines genome editing with in vitro genomic selection as considered by Visscher et al. [70], and by Goddard and Hayes [71]. One alternative that was modelled by Bastiaansen et al. [33], is to apply editing to all the offspring of elite individuals. In that case, the number of edits needed would be multiplied by the average number of offspring per sire. In any case, RAGE will require the development of advanced reproductive techniques, and it will be necessary to evaluate their use with empirical data from both the economic, ethical and animal welfare perspectives.

### Implications for breeding

We found that genome editing of deleterious alleles reduced deleterious load, but that when variants were recessive, simultaneous editing of multiple deleterious variants in the same sire was necessary for the approach to be competitive with selection against carriers. When accurate multiplex genome editing becomes available, RAGE will have the potential to improve fitness to levels that are impossible to attain by selection against carriers. This is a formidable undertaking, but a possible long-term goal. The long-term benefits of genome editing to remove deleterious variants over selection against carriers include both the possibility of higher gains in fitness, and the ability to improve fitness without sacrificing selection intensity for the breeding goal trait.

In the short-term, selection against carriers based on their total deleterious load is a possible alternative to genome editing. It is ineffective against codominant variants, but when variants are recessive, it is more effective at alleviating deleterious load than editing one variant per sire, but it is less effective than multiplex editing. The cost of multiplex genome editing is unknown, but it is assumed high. Therefore, it appears that selection against carriers will remain superior for some time. The downside of selection against carriers is that the number of sires available for selection is reduced, with associated risks of inbreeding and loss of genetic variation. Van Eenennaam and Kinghorn [72] and Cole [34, 73] have extended mate selection schemes to penalize the use of carrier animals or to prevent matings between carriers. It is possible that such methods could be extended to use genome-wide deleterious load while maintaining diversity in other parts of the genome and maximizing the response to selection for production traits.

To perform selection against carriers in practice, it will be necessary to include deleterious load in the selection index and to give it an economic weight to balance it with the breeding goal, and make sure that selection against deleterious load does not affect other traits unfavorably. Unfavorable correlations between estimated deleterious load and estimated breeding values for traits could arise either from false positives, pleiotropy, or linkage disequilibrium. Deleterious variant prediction methods may mistakenly classify beneficial variants as deleterious because they change protein function. In fact, they may have been even deleterious in the wild, but beneficial in a modern farm environment, such as the loss-of-function mutations in *myostatin* [74] that cause double muscling in beef cattle breeds. Deleterious variants may also have pleiotropic effects, as is the case with several recessive lethal haplotypes that are found at unexpectedly high frequencies in cattle breeds [75, 76]. In all these cases, it may be possible to use marker estimates from genomic selection models to prune the set of deleterious variants associated with large beneficial effects on other traits before calculating deleterious load.

## Conclusions

When accurate multiplex genome editing becomes available, removal of alleles by genome editing has the potential to improve fitness to levels that are impossible by selection against carriers. This is a formidable undertaking, but a possible long-term goal. RAGE requires simultaneous editing of multiple deleterious variants in the same sire to be effective. Priorities in the development of RAGE should be safe and accurate multiplex genome editing, and gathering large whole-genome sequencing datasets to estimate deleterious allele frequencies, deleterious load, and the correlations of deleterious load with traits under selection. Our results suggest that, in the future, there is potential to use RAGE against deleterious load to improve fitness traits in animal breeding populations.

## Additional files

**Additional file 1: Table S1.** Fitness, deleterious load, and number of segregating variants for different scenarios. Mean and standard deviation of fitness, deleterious load, and number of segregating variants for codominant (h = 0.5) and recessive (h = 0) variants with 5000, 10,000, and 15,000 of fitness variants in the genome, breeding goal traits drawn from a gamma distribution rather than a normal distribution, a shorter historical breeding (10 generations of natural selection and 5 generations of historical breeding), a longer historical breeding (25 generations), or a simpler population history (constant effective population size of 100). The numbers are based on 10 replicates.

**Additional file 2: Figure S1.** Distribution of deleterious allele frequencies and effects. Distributions of deleterious allele frequencies, and deleterious effect sizes for codominant (h = 0.5) and recessive (h = 0) variants with 5000, 10,000, and 15,000 of fitness variants in the genome, breeding goal traits drawn from a gamma distribution rather than a normal distribution, a shorter historical breeding (10 generations of natural selection and 5 generations of historical breeding), a longer historical breeding (25 generations), or a simpler population history (constant effective population size of 100).

**Additional file 3: Figure S2.** Deleterious load for different scenarios. Deleterious load, broken down in heterozygous and homozygous load, for codominant (h = 0.5) and recessive (h = 0) variants with 5000, 10,000, and 15,000 of fitness variants in the genome, breeding goal traits drawn from a gamma distribution rather than a normal distribution, a shorter historical breeding (10 generations of natural selection and 5 generations of historical breeding), a longer historical breeding (25 generations), or a simpler population history (constant effective population size of 100).

**Additional file 4: Figure S3.** Effect of selection against carriers. Average fitness over ten generations of future breeding with different selection strategies, and avoiding 100, 250, or 500 males when choosing sires. The discovery rate was 0.75. The lines show the average across 50 replicates.

## References

[1] Haldane JBS (1937). The effect of variation of fitness. Am Nat.

[2] Eyre-Walker A, Keightley PD (1999). High genomic deleterious mutation rates in hominids. Nature.

[3] Nachman MW, Crowell SL (2000). Estimate of the mutation rate per nucleotide in humans. Genetics.

[4] Kondrashov AS, Crow JF (1993). A molecular approach to estimating the human deleterious mutation rate. Hum Mutat.

[5] Charlier C, Coppieters W, Rollin F, Desmecht D, Agerholm JS, Cambisano N (2008). Highly effective SNP-based association mapping and management of recessive defects in livestock. Nat Genet.

[6] VanRaden PM, Olson KM, Null DJ, Hutchison JL (2011). Harmful recessive effects on fertility detected by absence of homozygous haplotypes. J Dairy Sci.

[7] Sahana G, Nielsen US, Aamand GP, Lund MS, Guldbrandtsen B (2013). Novel harmful recessive haplotypes identified for fertility traits in nordic holstein cattle. PLoS One.

[8] Fritz S, Capitan A, Djari A, Rodriguez SC, Barbat A, Baur A (2013). Detection of haplotypes associated with prenatal death in dairy cattle and identification of deleterious mutations in *GART*, *SHBG* and *SLC37A2*. PLoS One.

[9] Sonstegard TS, Cole JB, VanRaden PM, Van Tassell CP, Null DJ, Schroeder SG (2013). Identification of a nonsense mutation in *CWC15* associated with decreased reproductive efficiency in Jersey cattle. PLoS One.

[10] Flisikowski K, Venhoranta H, Nowacka-Woszuk J, McKay SD, Flyckt A, Taponen J (2010). A novel mutation in the maternally imprinted PEG3 domain results in a loss of MIMT1 expression and causes abortions and stillbirths in cattle (*Bos taurus*). PLoS One.

[11] Schütz E, Wehrhahn C, Wanjek M, Bortfeld R, Wemheuer WE, Beck J (2016). The Holstein Friesian lethal haplotype 5 (HH5) results from a complete deletion of *TBF1M* and cholesterol deficiency (CDH) from an ERV-(LTR) insertion into the coding region of *APOB*. PLoS One.

[12] Derks MFL, Megens HJ, Bosse M, Lopes MS, Harlizius B, Groenen MAM (2017). A systematic survey to identify lethal recessive variation in highly managed pig populations. BMC Genomics.

[13] Boyko AR, Williamson SH, Indap AR, Degenhardt JD, Hernandez RD, Lohmueller KE (2008). Assessing the evolutionary impact of amino acid mutations in the human genome. PLoS Genet.

[14] Eyre-Walker A, Woolfit M, Phelps T (2006). The distribution of fitness effects of new deleterious amino acid mutations in humans. Genetics.

[15] Loewe L, Charlesworth B (2006). Inferring the distribution of mutational effects on fitness in Drosophila. Biol Lett.

[16] Keightley PD, Eyre-Walker A (2007). Joint inference of the distribution of fitness effects of deleterious mutations and population demography based on nucleotide polymorphism frequencies. Genetics.

[17] Davydov EV, Goode DL, Sirota M, Cooper GM, Sidow A, Batzoglou S (2010). Identifying a high fraction of the human genome to be under selective constraint using GERP++. PLoS Comput Biol.

[18] Pejaver V, Urresti J, Lugo-Martinez J, Pagel KA, Lin GN, Nam H-J (2017). MutPred2: inferring the molecular and phenotypic impact of amino acid variants. BioRxiv.

[19] Siepel A, Bejerano G, Pedersen JS, Hinrichs AS, Hou M, Rosenbloom K (2005). Evolutionarily conserved elements in vertebrate, insect, worm, and yeast genomes. Genome Res.

[20] Ng PC, Henikoff S (2003). SIFT: predicting amino acid changes that affect protein function. Nucleic Acids Res.

[21] Adzhubei IA, Schmidt S, Peshkin L, Ramensky VE, Gerasimova A, Bork P (2010). A method and server for predicting damaging missense mutations. Nat Methods.

[22] Ramu P, Esuma W, Kawuki R, Rabbi IY, Egesi C, Bredeson JV (2017). Cassava haplotype map highlights fixation of deleterious mutations during clonal propagation. Nat Genet.

[23] Mezmouk S, Ross-Ibarra J (2014). The pattern and distribution of deleterious mutations in maize. G3 (Bethesda).

[24] Yang J, Mezmouk S, Baumgarten A, Buckler ES, Guill KE, McMullen MD (2017). Incomplete dominance of deleterious alleles contributes substantially to trait variation and heterosis in maize. PLoS Genet.

[25] Bianco E, Nevado B, Ramos-Onsins SE, Pérez-Enciso M (2015). A deep catalog of autosomal single nucleotide variation in the pig. PLoS One.

[26] Daetwyler HD, Capitan A, Pausch H, Stothard P, Van Binsbergen R, Brøndum RF (2014). Whole-genome sequencing of 234 bulls facilitates mapping of monogenic and complex traits in cattle. Nat Genet.

[27] Das A, Panitz F, Gregersen VR, Bendixen C, Holm LE (2015). Deep sequencing of Danish Holstein dairy cattle for variant detection and insight into potential loss-of-function variants in protein coding genes. BMC Genomics.

[28] Kircher M, Witten DM, Jain P, O’Roak BJ, Cooper GM, Shendure J (2014). A general framework for estimating the relative pathogenicity of human genetic variants. Nat Genet.

[29] Liu X, Wu C, Li C, Boerwinkle E (2016). dbNSFP v3. 0: a one-stop database of functional predictions and annotations for human nonsynonymous and splice-site SNVs. Hum Mutat.

[30] Gaj T, Gersbach CA, Barbas CF (2013). ZFN, TALEN, and CRISPR/Cas-based methods for genome engineering. Trends Biotechnol.

[31] Capecchi MR (2005). Gene targeting in mice: functional analysis of the mammalian genome for the twenty-first century. Nat Rev Genet.

[32] Jenko J, Gorjanc G, Cleveland MA, Varshney RK, Whitelaw CBA, Woolliams JA (2015). Potential of promotion of alleles by genome editing to improve quantitative traits in livestock breeding programs. Genet Sel Evol.

[33] Bastiaansen JWM, Bovenhuis H, Groenen MAM, Megens HJ, Mulder HA (2018). The impact of genome editing on the introduction of monogenic traits in livestock. Genet Sel Evol.

[34] Cole JB (2017). Management of Mendelian traits in breeding programs by gene editing: a simulation study. BioRxiv.

[35] Sonesson AK, Janss LLG, Meuwissen THE (2003). Selection against genetic defects in conservation schemes while controlling inbreeding. Genet Sel Evol.

[36] Chen GK, Marjoram P, Wall JD (2009). Fast and flexible simulation of DNA sequence data. Genome Res.

[37] Hayes B, Goddard ME (2001). The distribution of the effects of genes affecting quantitative traits in livestock. Genet Sel Evol.

[38] Core Team R (2017). R: a language and environment for statistical computing.

[39] Eddelbuettel D, François R (2011). Rcpp: seamless R and C++ integration. J Stat Softw.

[40] Eddelbuettel D, Sanderson C (2014). RcppArmadillo: accelerating R with high-performance C++ linear algebra. Comput Stat Data Anal.

[41] Sanderson C, Curtin R (2016). Armadillo: a template-based C++ library for linear algebra. J Open Source Softw.

[42] Wickham H (2016). ggplot2: elegant graphics for data analysis.

[43] MacArthur DG, Balasubramanian S, Frankish A, Huang N, Morris J, Walter K (2012). A systematic survey of loss-of-function variants in human protein-coding genes. Science.

[44] Lek M, Karczewski KJ, Minikel EV, Samocha KE, Banks E, Fennell T (2016). Analysis of protein-coding genetic variation in 60,706 humans. Nature.

[45] Abecasis GR, Auton A, Brooks LD, DePristo MA, Durbin RM, Consortium 1000 Genomes Project (2012). An integrated map of genetic variation from 1,092 human genomes. Nature.

[46] Charlier C, Li W, Harland C, Littlejohn M, Coppieters W, Creagh F (2016). NGS-based reverse genetic screen for common embryonic lethal mutations compromising fertility in livestock. Genome Res.

[47] Chun S, Fay JC (2009). Identification of deleterious mutations within three human genomes. Genome Res.

[48] Tennessen JA, Bigham AW, O’Connor TD, Fu W, Kenny EE, Gravel S (2012). Evolution and functional impact of rare coding variation from deep sequencing of human exomes. Science.

[49] Bosse M, Megens HJ, Madsen O, Crooijmans RPMA, Ryder OA, Austerlitz F (2015). Using genome-wide measures of coancestry to maintain diversity and fitness in endangered and domestic pig populations. Genome Res.

[50] Falconer DS, Mackay TFC (1996). Introduction to quantitative genetics.

[51] Agrawal AF, Whitlock MC (2011). Inferences about the distribution of dominance drawn from yeast gene knockout data. Genetics.

[52] Mukai T, Chigusa SI, Mettler LE, Crow JF (1972). Mutation rate and dominance of genes affecting viability in *Drosophila melanogaster*. Genetics.

[53] Houle D, Hughes KA, Assimacopoulos S, Charlesworth B (1997). The effects of spontaneous mutation on quantitative traits. II. Dominance of mutations with effects on life-history traits. Genet Res.

[54] Charlesworth D, Willis JH (2009). The genetics of inbreeding depression. Nat Rev Genet.

[55] Leroy G (2014). Inbreeding depression in livestock species: review and meta-analysis. Anim Genet.

[56] Jao LE, Wente SR, Chen W (2013). Efficient multiplex biallelic zebrafish genome editing using a CRISPR nuclease system. Proc Natl Acad Sci USA.

[57] Ousterout DG, Kabadi AM, Thakore PI, Majoros WH, Reddy TE, Gersbach CA (2015). Multiplex CRISPR/Cas9-based genome editing for correction of dystrophin mutations that cause Duchenne muscular dystrophy. Nat Commun.

[58] Niu D, Wei HJ, Lin L, George H, Wang T, Lee IH (2017). Inactivation of porcine endogenous retrovirus in pigs using CRISPR-Cas9. Science.

[59] Wang H, Yang H, Shivalila CS, Dawlaty MM, Cheng AW, Zhang F (2013). One-step generation of mice carrying mutations in multiple genes by CRISPR/Cas-mediated genome engineering. Cell.

[60] González F, Zhu Z, Shi ZD, Lelli K, Verma N, Li QV (2014). An iCRISPR platform for rapid, multiplexable, and inducible genome editing in human pluripotent stem cells. Cell Stem Cell.

[61] McLaren W, Gil L, Hunt SE, Riat HS, Ritchie GRS, Thormann A (2016). The ensembl variant effect predictor. Genome Biol.

[62] Cingolani P, Platts A, Wang LL, Coon M, Nguyen T, Wang L (2012). A program for annotating and predicting the effects of single nucleotide polymorphisms, SnpEff: SNPs in the genome of Drosophila melanogaster strain w1118; iso-2; iso-3. Fly (Austin).

[63] Quang D, Chen Y, Xie X (2015). DANN: a deep learning approach for annotating the pathogenicity of genetic variants. Bioinformatics.

[64] Zhou J, Troyanskaya OG (2015). Predicting effects of noncoding variants with deep learning-based sequence model. Nat Methods.

[65] Eőry L, Halligan DL, Keightley PD (2009). Distributions of selectively constrained sites and deleterious mutation rates in the hominid and murid genomes. Mol Biol Evol.

[66] Keightley PD, Gaffney DJ (2003). Functional constraints and frequency of deleterious mutations in noncoding DNA of rodents. Proc Nat Acad Sci USA.

[67] Hill WG, Robertson A (1966). The effect of linkage on limits to artificial selection. Genet Res.

[68] Rodgers-Melnick E, Bradbury PJ, Elshire RJ, Glaubitz JC, Acharya CB, Mitchell SE (2015). Recombination in diverse maize is stable, predictable, and associated with genetic load. Proc Natl Acad Sci USA.

[69] Bernardo R (2017). Prospective targeted recombination and genetic gains for quantitative traits in maize. Plant Genome.

[70] Visscher P, Pong-Wong R, Whittemore C, Haley C (2000). Impact of biotechnology on (cross) breeding programmes in pigs. Livest Prod Sci.

[71] Goddard ME, Hayes BJ (2007). Genomic selection. J Anim Breed Genet.

[72] Van Eenennaam AL, Kinghorn BP (2014) Use of mate selection software to manage lethal recessive conditions in livestock populations. In: Proceedings of the 10th world congress on genetics applied to livestock production: 17–22 Aug 2014. Vancouver.

[73] Cole JB (2015). A simple strategy for managing many recessive disorders in a dairy cattle breeding program. Genet Sel Evol.

[74] Dunner S, Miranda ME, Amigues Y, Cañón J, Georges M, Hanset R (2003). Haplotype diversity of the *myostatin* gene among beef cattle breeds. Genet Sel Evol.

[75] Cole JB, Null DJ, VanRaden PM (2016). Phenotypic and genetic effects of recessive haplotypes on yield, longevity, and fertility. J Dairy Sci.

[76] Jenko J, McClure MC, Matthews D, McClure J, Johnsson M, Gorjanc G (2019). Analysis of a large data set reveals haplotypes carrying putatively recessive lethal alleles with pleiotropic effects on economically important traits in beef cattle. Genet Sel Evol.

