# Supplementary material for: Removal of alleles by genome editing (RAGE) against deleterious load
Source: Genet Sel Evol. 2019 Apr 17;51:14. doi: 10.1186/s12711-019-0456-8 (PMC6472060; doi:10.1186/s12711-019-0456-8)
Supplement: Supplementary file 1 — Additional file 1: Table S1. Fitness, deleterious load, and number of segregating variants for different scenarios. Mean and standard deviation of fitness, deleterious load, and number of segregating variants for codominant (h = 0.5) and recessive (h = 0) variants with 5000, 10,000, and 15,000 of fitness variants in the genome, breeding goal traits drawn from a gamma distribution rather than a normal distribution, a shorter historical breeding (10 generations of natural selection and 5 generations of historical breeding), a longer historical breeding (25 generations), or a simpler population history (constant effective population size of 100). The numbers are based on 10 replicates. [file 12711_2019_456_MOESM1_ESM.pdf]

Additional file 1. Mean and standard deviation of fitness, deleterious load, and number of segregating variants for codominant (h = 0.5) and recessive (h = 0) variants with 5000, 10 000, and 15 000 of fitness variants in the genome, breeding goal traits drawn from a gamma distribution rather than a normal distribution, a shorter historical breeding (10 generations of natural selection and 5 generations of historical breeding), a longer historical breeding (25 generations), or a simpler population history (constant effective population size of 100). The numbers are based on 10 replicates.

Codominant variants

Scenario	Fitness	s.d.	Load	s.d.	Segregating variants	s.d.
10000 variants	0.58	0.02	52.32	7.63	4444	217
15000 variants	0.60	0.05	53.64	7.76	4730	476
5000 variants	0.57	0.03	48.04	7.16	4347	342
gamma effects	0.58	0.03	52.35	8.40	4542	271
long historical	0.59	0.03	57.37	8.04	4534	376
short historical	0.66	0.04	22.50	4.86	4966	328
simple history	0.56	0.06	50.85	7.20	4534	564

Recessive variants

Scenario	Fitness	s.d.	Load	s.d.	Segregating variants	s.d.
10000 variants	0.86	0.02	88.75	9.66	3634	208
15000 variants	0.81	0.03	109.20	10.55	3222	218
5000 variants	0.87	0.02	76.93	8.84	3727	177
gamma effects	0.82	0.04	96.50	10.76	3283	350
long historical	0.81	0.06	101.27	10.63	3351	364
short historical	0.97	0.00	56.92	7.94	4631	63
simple history	0.88	0.02	78.12	9.55	3351	314
