# Supplementary figures and images for: Removal of alleles by genome editing (RAGE) against deleterious load
Source: Genet Sel Evol. 2019 Apr 17;51:14. doi: 10.1186/s12711-019-0456-8 (PMC6472060; doi:10.1186/s12711-019-0456-8)

## Codominant

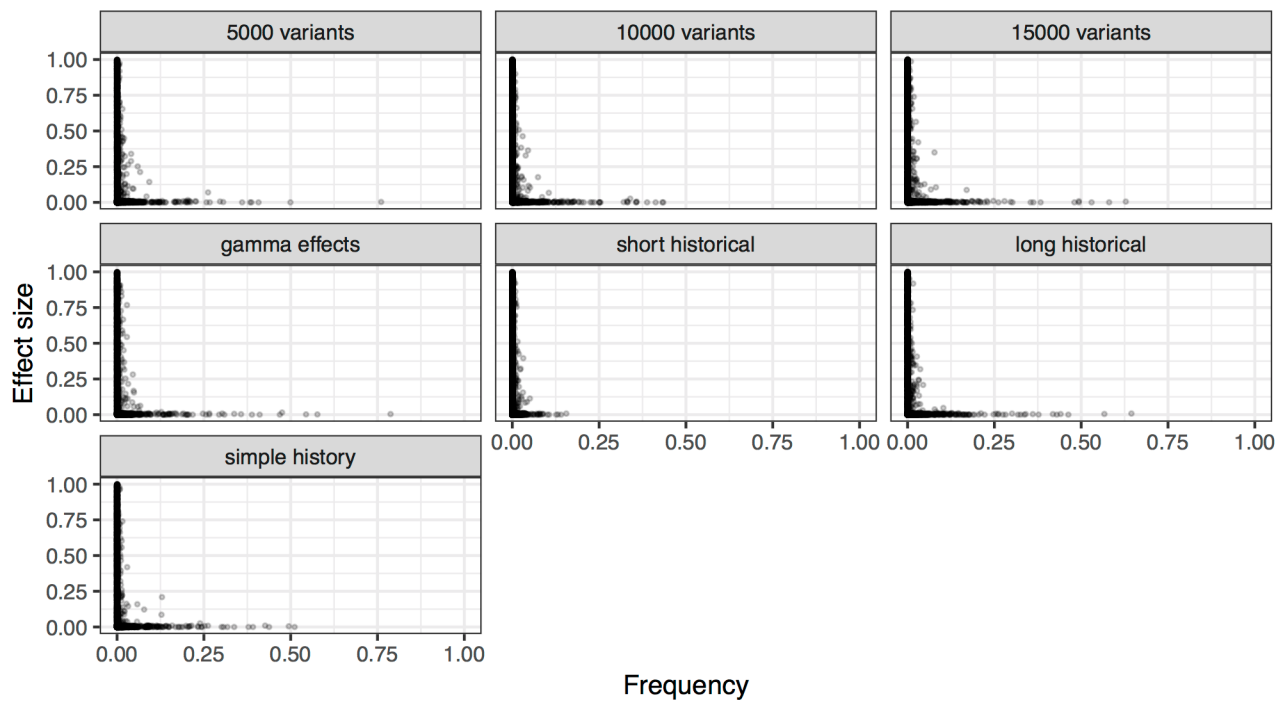

## Recessive

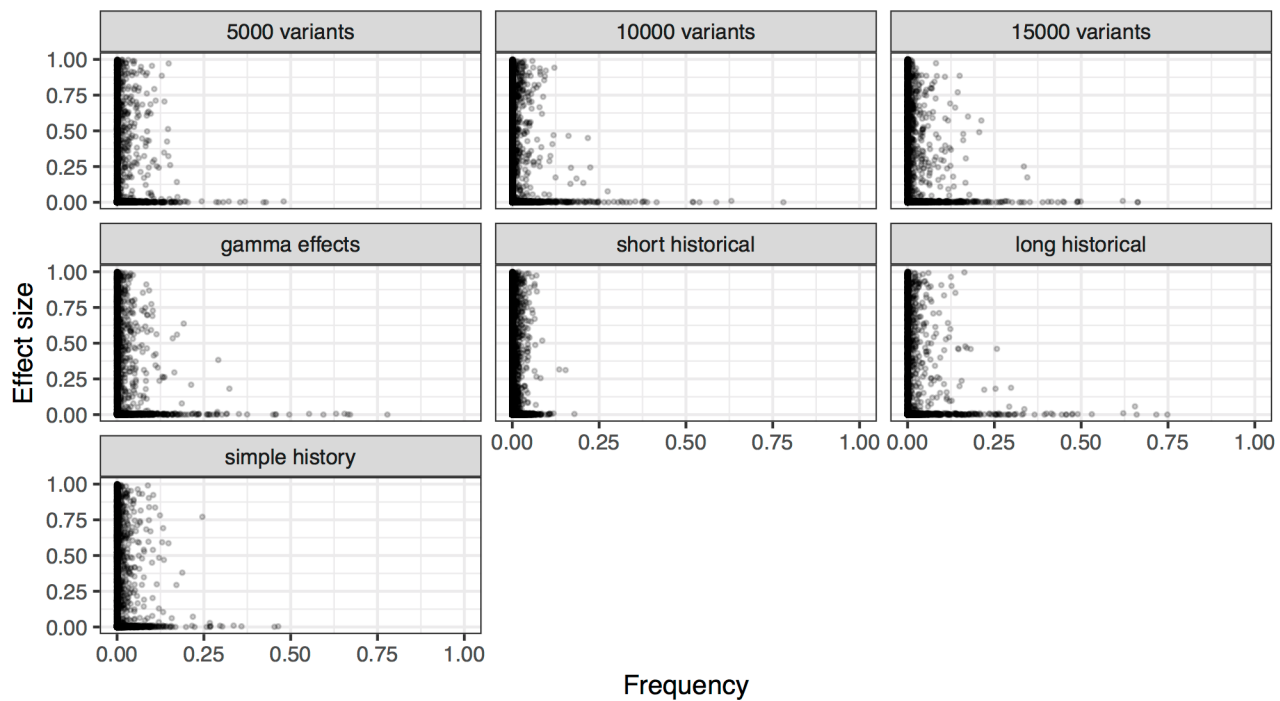

Supplement: Supplementary file 2 — Additional file 2: Figure S1. Distribution of deleterious allele frequencies and effects. Distributions of deleterious allele frequencies, and deleterious effect sizes for codominant (h = 0.5) and recessive (h = 0) variants with 5000, 10,000, and 15,000 of fitness variants in the genome, breeding goal traits drawn from a gamma distribution rather than a normal distribution, a shorter historical breeding (10 generations of natural selection and 5 generations of historical breeding), a longer historical breeding (25 generations), or a simpler population history (constant effective population size of 100). [file 12711_2019_456_MOESM2_ESM.pdf]

# Codominant

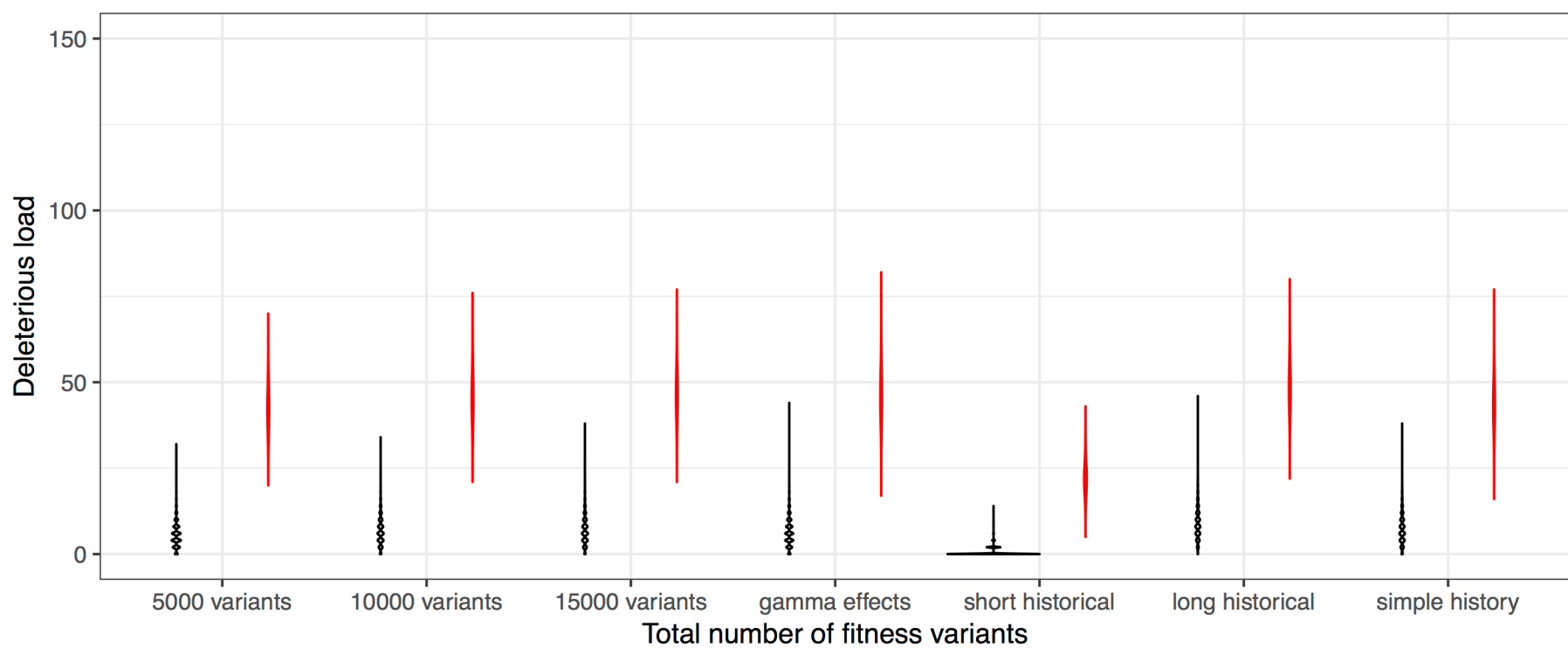

# Recessive

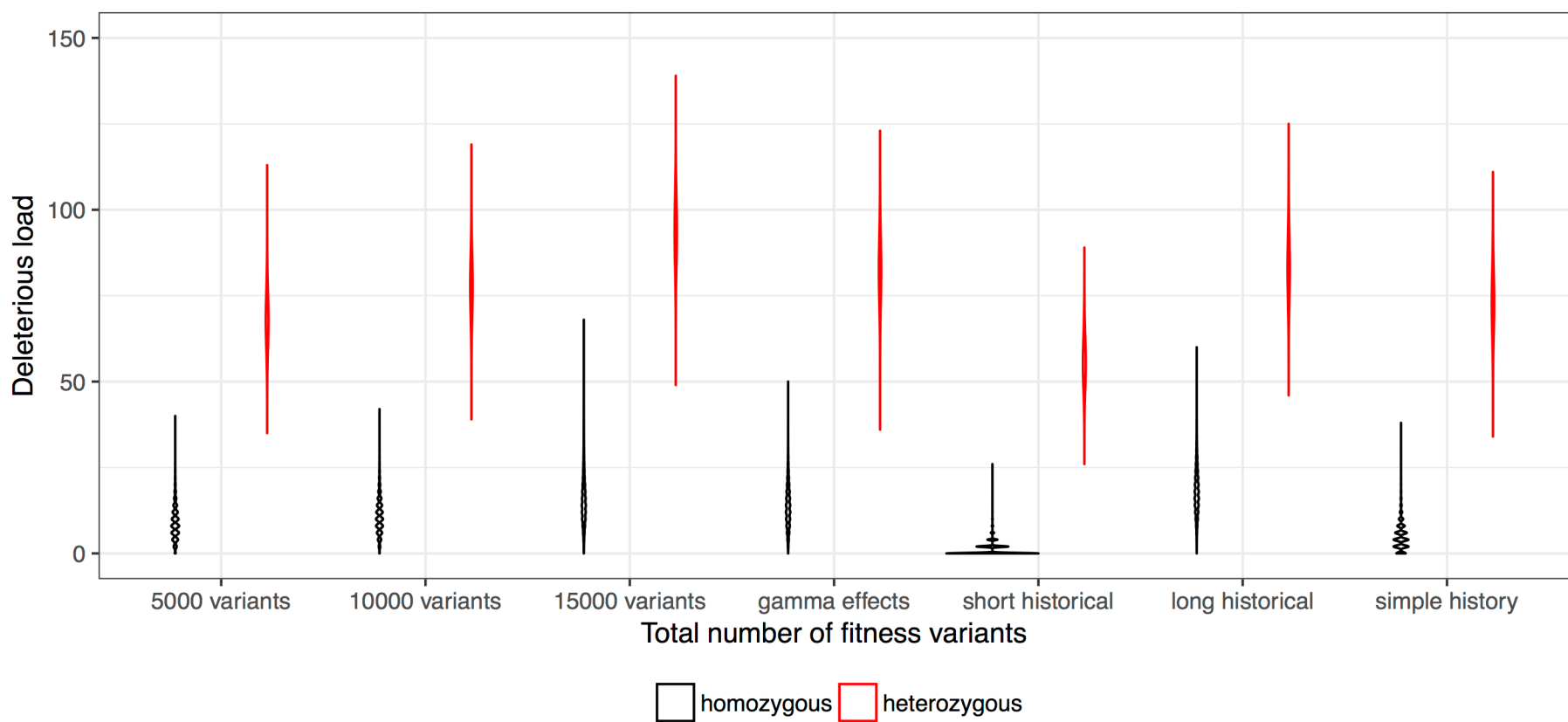

Supplement: Supplementary file 3 — Additional file 3: Figure S2. Deleterious load for different scenarios. Deleterious load, broken down in heterozygous and homozygous load, for codominant (h = 0.5) and recessive (h = 0) variants with 5000, 10,000, and 15,000 of fitness variants in the genome, breeding goal traits drawn from a gamma distribution rather than a normal distribution, a shorter historical breeding (10 generations of natural selection and 5 generations of historical breeding), a longer historical breeding (25 generations), or a simpler population history (constant effective population size of 100). [file 12711_2019_456_MOESM3_ESM.pdf]

## Codominant

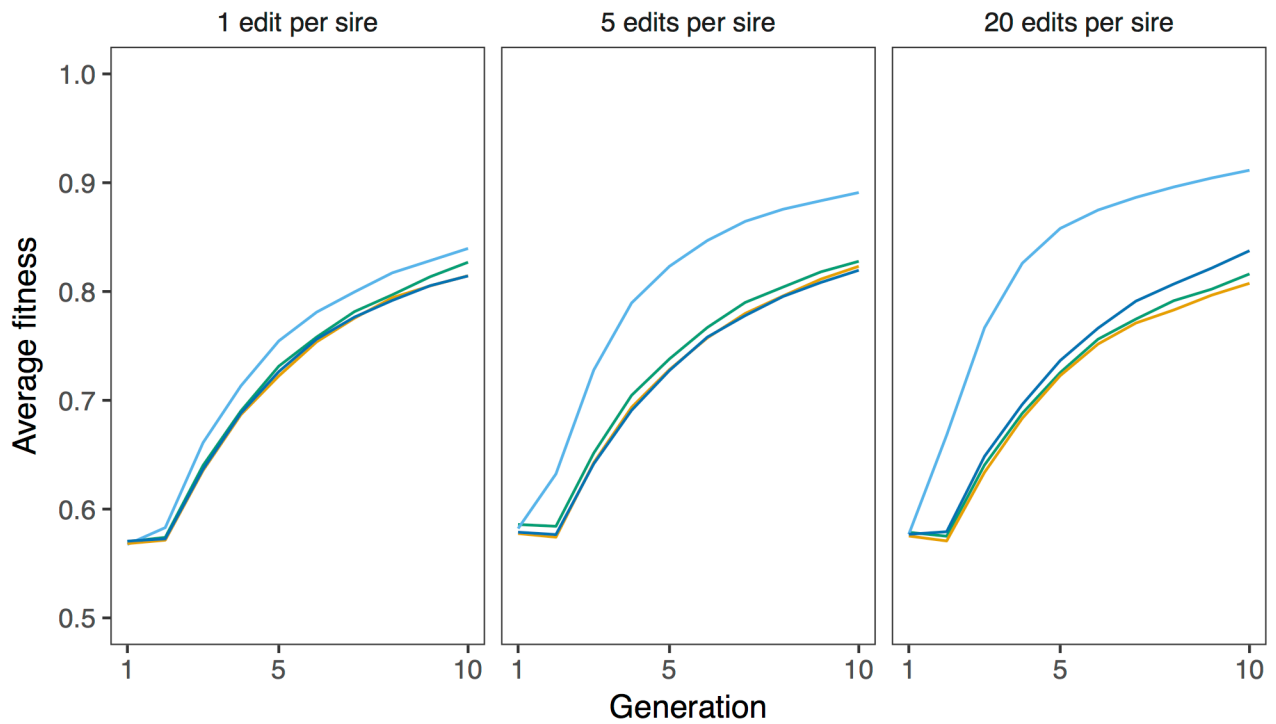

## Recessive

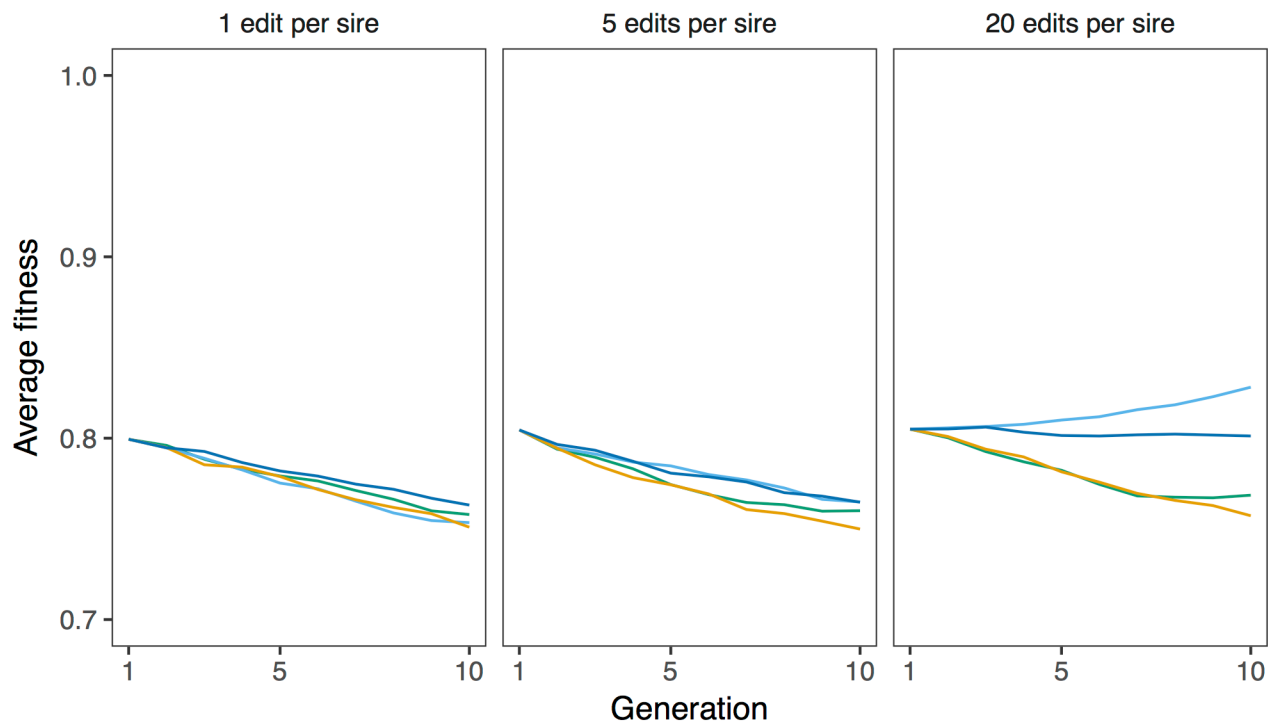

— no editing — low frequency first — high frequency first — random order

Supplement: Supplementary file 4 — Additional file 4: Figure S3. Effect of selection against carriers. Average fitness over ten generations of future breeding with different selection strategies, and avoiding 100, 250, or 500 males when choosing sires. The discovery rate was 0.75. The lines show the average across 50 replicates. [file 12711_2019_456_MOESM4_ESM.pdf]
